# Editorial: Bone and muscle interactions in bone pathologies

**DOI:** 10.3389/fendo.2023.1326694

**Published:** 2023-11-07

**Authors:** Trupti Trivedi, Mark Hamrick

**Affiliations:** ^1^ Department of Endocrine Neoplasia and Hormonal Disorders, University of Texas, MD Anderson Cancer Center, Houston, TX, United States; ^2^ Medical College of Georgia, Augusta University, Augusta, GA, United States

**Keywords:** pathologic bone destruction, musculoskeletal, bone resorption, bone-targeted therapies, bone marrow adiposity

Bone and muscle are tightly integrated tissues which provide balance and functional support by mechanical and molecular interactions. Active research is underway to understand the mechanisms of bone and muscle crosstalk which can have therapeutic advantages to treat musculoskeletal diseases. Interaction of bone-derived factors with muscle function is an evolving area of research. Similarly, muscle weakness either due to loss of muscle mass or due to loss of muscle function or combination of both can dysregulate physiological balance in bone cells. Cancer cachexia, fatty infiltration in muscle, lack of exercise, sarcopenia, muscle injury, and hormonal imbalances can cause abnormal release of muscle-derived actors. These factors can, in turn, interfere with bone remodeling process leading to pathological bone destruction. If excess bone resorption is associated with muscle weakness, then it may increase fracture risk, reducing the therapeutic response of currently available bone-targeted therapeutics, negatively impacting health outcomes. Thus, it is critical to understand the interaction between bone and muscle. Similarly, it is important to identify the mechanisms of bone loss in the settings of muscle dysfunction.

We compiled original research articles submitted to the present Research Topic of Frontiers in Endocrinology. These novel contributions have added important new perspectives to our understanding of bone and muscle interaction. Published articles have covered molecular interactions between bone and muscle. For example, the seminal contribution from Hong et al. found that in patients with osteoporosis, maintenance of muscle mass and function would be influenced more by muscle GDF-15 than by circulating GDF-15. The authors have shown that serum GDF-15 is higher in the osteoporosis group than control group, and that serum GDF-15 was positively correlated with age and negatively with BMD (Bone Mineral Density) of hip. Additionally, muscle GDF-15 concentrations but not serum GDF-15 were correlated with handgrip power or circumferences of the upper arm and calf. The study conducted by Guo et al., revealed that hydrogen saline water treatment in ovariectomized rats suppressed autophagy and then activated Keap1-Nrf2 signaling pathway by maintaining the Keap1-Nrf2-P62 interaction. This treatment also improved osteoporotic fracture healing process.

The present Research Topic also covers the utility of different imaging modalities in understanding the crosstalk among bone, muscle, and fat. Zhang et al. reported that in young to middle age male patients with Crohn’s disease total, visceral and subcutaneous adipose tissue mass were reduced, and this reduction was associated with reduced BMD. The authors have highlighted that the area of lean mass on the abdomen and muscle mass around lumbar vertebrae was also reduced in patients with Crohn’s disease, which could be associated with reduced BMD. Their paper concluded by highlighting the importance of considering protection of bone health while making treatment decisions for the patients with Crohn’s diseases. Badr et al. explored the interaction among muscle, fat, and bone by evaluating the role of intramuscular fat (myosteatosis) and bone marrow adiposity in postmenopausal woman with or without a fragility fracture. The authors observed that, despite a higher intramuscular fatty infiltration in the fracture group, there was no relation between bone marrow adiposity and myosteatosis in patients with fragility fractures. Although myosteatosis was not associated with bone marrow fat it was associated with visceral adipose tissue and total body fat. The authors concluded that bone marrow adiposity is a unique fat depot with a weak relationship to other white adipose tissues. Using a cross-sectional study of older Asian adults, Liu et al. determined the importance of muscle mass versus fat content in prevention of osteoporosis. The risk of osteoporosis in older individuals with obesity or sarcopenia was estimated using regression analysis. Their study concluded by recommending that in older people, bone health may benefit more from improving muscle mass and strength rather than by controlling obesity. Using two-sample mendelian randomization (MR) analysis, Lin et al. explored the relationship between osteoporosis and osteoarthritis and predicated that osteoporosis can reduce the incidence of OA.

Qiu et al. performed an elegant predictive study to identify the diagnostic efficacy of the paravertebral muscle group in type 2 diabetes patients as a novel strategy for the diagnosis and treatment of osteoporosis. The authors used a radiomics approach with sophisticated data analysis tools for the predication of vertebral fracture risk using paravertebral muscle as a predictor of fracture risk in type 2 diabetes. Using a combination model of nomogram and radiomics, it was concluded that the radiomics features for paraspinal muscle group have a superior performance in predicating changes in bone mass in T2DM which can be a better method for making clinical decisions.

Together, the information provided by the original research articles submitted to this Research Topic expand significantly upon our current understanding of bone, muscle, and fat interactions. Despite these contributions, there are still some unexplored areas that need more attention in the direction of therapeutic development and improvement of musculoskeletal outcome in patients suffering from debilitating skeletal related events.

## Author contributions

TT: Conceptualization, Writing – original draft, Writing – review & editing. MH: Writing – review & editing.

